# Multiscale Analysis of Metal Oxide Nanoparticles in Tissue: Insights into Biodistribution and Biotransformation

**DOI:** 10.1002/advs.202000912

**Published:** 2020-06-18

**Authors:** Martin T. Matter, Jian‐Hao Li, Ioana Lese, Claudia Schreiner, Laetitia Bernard, Olivier Scholder, Jasmin Hubeli, Kerda Keevend, Elena Tsolaki, Enrico Bertero, Sergio Bertazzo, Robert Zboray, Radu Olariu, Mihai A. Constantinescu, Renato Figi, Inge K. Herrmann

**Affiliations:** ^1^ Particles‐Biology Interactions, Department of Materials Meet Life Swiss Federal Laboratories for Materials Science and Technology (Empa) Lerchenfeldstrasse 5 St. Gallen 9014 Switzerland; ^2^ Nanoparticle Systems Engineering Laboratory Institute of Process Engineering Department of Mechanical and Process Engineering ETH Zurich Sonneggstrasse 3 Zurich 8092 Switzerland; ^3^ Department of Plastic and Hand Surgery University Hospital Bern (Inselspital) University of Bern Bern 3010 Switzerland; ^4^ Advanced Analytical Technologies Swiss Federal Laboratories for Materials Science and Technology (Empa) Uberlandstrasse 129 Dubendorf 8600 Switzerland; ^5^ Nanoscale Materials Department of Materials Meet Life Swiss Federal Laboratories for Materials Science and Technology (Empa) Uberlandstrasse 129 Dubendorf 8600 Switzerland; ^6^ Department of Medical Physics and Biomedical Engineering University College London (UCL) Malet Place Engineering Building London WC1E 6BT UK; ^7^ Mechanics of Materials and Nanostructures Swiss Federal Laboratories for Materials Science and Technology (Empa) Feuerwerkerstrasse 39 Thun 3602 Switzerland; ^8^ Center for X‐ray Analytics Swiss Federal Laboratories for Materials Science and Technology (Empa) Uberlandstrasse 129 Dubendorf 8600 Switzerland

**Keywords:** analytical imaging, biological fate, multiscale, nanosafety, spectral unmixing

## Abstract

Metal oxide nanoparticles have emerged as exceptionally potent biomedical sensors and actuators due to their unique physicochemical features. Despite fascinating achievements, the current limited understanding of the molecular interplay between nanoparticles and the surrounding tissue remains a major obstacle in the rationalized development of nanomedicines, which is reflected in their poor clinical approval rate. This work reports on the nanoscopic characterization of inorganic nanoparticles in tissue by the example of complex metal oxide nanoparticle hybrids consisting of crystalline cerium oxide and the biodegradable ceramic bioglass. A validated analytical method based on semiquantitative X‐ray fluorescence and inductively coupled plasma spectrometry is used to assess nanoparticle biodistribution following intravenous and topical application. Then, a correlative multiscale analytical cascade based on a combination of microscopy and spectroscopy techniques shows that the topically applied hybrid nanoparticles remain at the initial site and are preferentially taken up into macrophages, form apatite on their surface, and lead to increased accumulation of lipids in their surroundings. Taken together, this work displays how modern analytical techniques can be harnessed to gain unprecedented insights into the biodistribution and biotransformation of complex inorganic nanoparticles. Such nanoscopic characterization is imperative for the rationalized engineering of safe and efficacious nanoparticle‐based systems.

## Graphical abstract

The development of novel nanotherapeutics requires an in‐depth understanding of their molecular interplay with biology. This multidisciplinary work elucidates the biodistribution, biotransformation, and local biological impact of complex inorganic nanoparticles using a correlative cascade of advanced elemental analysis, label‐free microscopy, and spectroscopy techniques. This work illustrates the potential of advanced analytical techniques to deepen the understanding of nanotherapeutics.

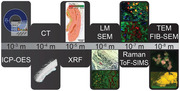

## 1 Introduction

Nanoparticle‐based materials are increasingly used in the biomedical field due to their versatility and exclusive properties. Nanoscale engineering has given rise to a whole new toolset for the design of innovative biomedical materials.^[^ ^1^ ^]^ It enables the bottom‐up synthesis of hierarchical structures with ultrahigh precision,^[^ ^2^ ^]^ which opens the possibility to directly engineer the interactions of structures with the surrounding environment across a wide range of length scales.^[^ ^3^ ^]^ However, steering the material‐biology interplay at the nanoscale implies a detailed understanding of the reactions occurring in the biological environment upon nanoparticle exposure and vice versa.^[^ ^4^ ^]^

Most recent developments in the biomedical field have shifted the focus from complex, often over‐engineered solutions to more robust, easy‐to‐use approaches.^[^ ^5^ ^]^ Based on this shift, many scientists have embraced metal and metal oxide nanoparticles for a wide spectrum of applications such as sensing, diagnosis, delivery, and as pharmacologically active agents.^[^ ^6^ ^]^ Such nanoparticles exhibit well‐documented direct and robust bioactivity, which does not rely on complex organic post‐modification. Metal and metal oxide nanoparticles have shown great potential as contrast agents for different medical imaging techniques,^[^ ^7^ ^]^ cancer treatment,^[^ ^8^ ^]^ and drug delivery.^[^ ^9^ ^]^ Metal oxide nanoparticles are especially relevant for the wound management field, where the advent of nanomaterials enabled easy‐to‐use solutions that eliminate the need for cross‐linking reactions or in vivo polymerization.^[^ ^10^ ^]^ This is best illustrated by a recent example where Leibler and colleagues^[^ ^11^ ^]^ have introduced the concept of nano‐bridging, which can be employed to achieve tissue adhesion by a mere physical phenomenon.^[^ ^12^ ^]^ Additionally to binding tissue, metal oxide nanoparticles also allow the incorporation of bioactive entities. While there is a variety of materials^[^ ^13^ ^]^ and ions^[^ ^14^ ^]^ of interest, especially bioactive ceramics have been shown to improve wound healing in numerous ways.^[^ ^15^ ^]^ Recently, the adhesive and bioactive properties of such nanoparticles have been combined in the development of ceria (CeO_2_)/bioglass (45S5, BG) hybrid nanoparticle‐based tissue glues. Topically applied aqueous suspensions of these nanoparticles have been shown to significantly increase the blood perfusion and the survival of full‐thickness skin flaps in a rat model by promoting adhesion and angiogenesis.^[^ ^16^ ^]^ Various advantages of such nano‐enabled solutions compared to current wound closing techniques have been demonstrated.^[^ ^12^ ^]^ In general, metal oxide nanoparticles are being researched intensely and show great potential in biomedicine, yet they have a difficult stand in terms of translation to clinics. While some metal oxide nanoparticles have received market approval,^[^ ^17^ ^]^ especially as imaging agents,^[^ ^18^ ^]^ their translation has been slow in recent years.^[^ ^19^, ^20^ ^]^ For many metal oxide nanosystems there are safety concerns regarding their poor degradability and the limited understanding of their in vivo fate.^[^ ^21^, ^22^ ^]^ The detailed understanding of the interactions of nanoparticles with biological entities, especially their redistribution and degradation, remains pivotal for the safe and efficacious development of nanoparticle‐based products in general.^[^ ^23^ ^]^ The inability to measure and assess this interplay renders both improvement and regulation of nanomedicine extremely challenging. These challenges are reflected in the low approval rates of nanomedicines, despite immense investments by academia and industry in recent years.^[^ ^19^, ^20^ ^]^

In contrast to the much‐studied intravascular application, topical application of adhesive metal oxide nanoparticle suspensions has the prospect to limit the re‐distribution of the nanoparticles to non‐target sites.^[^ ^24^ ^]^ Reaching such an understanding, however, is not straightforward, especially for a complex system such as the aforementioned hybrid, which contains a stable, crystalline (ceria) and a biodegradable, amorphous (BG) phase in one nanoparticulate entity.^[^ ^25^ ^]^ Current medical imaging technologies do not have the spatial resolution required to yield information on a single‐particle scale. Most studies that address in vivo nanoparticle fate use computer X‐ray tomography with relatively poor sensitivity or magnetic resonance imaging,^[^ ^26^ ^]^ that is limited at around 30 µm.^[^ ^27^ ^]^ Higher resolution analysis including histology, X‐ray and fluorescence imaging, are typically performed on ex vivo tissues.^[^ ^28^ ^]^ These techniques have typical resolutions of 10 µm (X‐ray microtomography, laser‐induced breakdown spectroscopy), or 1 µm in high‐resolution 2D micro X‐ray fluorescence (μXRF).^[^ ^29^ ^]^ Laser ablation inductively coupled plasma mass spectrometry (LA‐ICP‐MS) reaches high sensitivities of 500 ppb for heavy elements (e.g., Au), however, typical spot diameters are limited to 1–10 µm.^[^ ^29^ ^]^ Most of the methods with ultrahigh sensitivity are either based on transmission electron microscopy, which gives information about single‐particle distribution in the biological sample, but has a limited field of view, or on elemental analysis of the bulk and thus do not yield spatially resolved information.^[^ ^30^ ^]^ In other words, there is a sub‐micrometer localization gap for nanoparticles after in vivo application. Not only localization of the applied nanoparticles is important, but also the evaluation of their chemical environment and degradation. Physicochemical changes at the nanoscale, such as particle uptake and agglomeration, phase transformation, and degradation are pivotal, yet difficult to monitor.^[^ ^21^, ^22^, ^23^, ^24^, ^25^, ^26^, ^27^, ^28^, ^29^, ^30^, ^31^ ^]^

Here, we report on a nano‐analytical cascade that can bridge the nanoparticle localization gap and allows the assessment of their physicochemical properties and their local metabolic impact. We selected complex nanoparticles used as tissue adhesives as an analytically very challenging but medically relevant material to showcase the opportunities and limitations of the proposed analytical imaging cascade. These metal oxide nanoparticle hybrids consisting of bioglass and ceria (BG/ceria) were applied either topically as tissue glues to the subcutis in a rat skin flap model or systemically. First, we present the physicochemical characterization of the as‐prepared metal oxide hybrid nanoparticles (BG/ceria) prior to application. Following topical application in vivo, we then report on the micro‐ and nano‐analytical assessment of the particle fate and biotransformation by cutting‐edge analytical technologies. We present results on the compositional analysis of these complex nanoparticle hybrids along with biodistribution data at unprecedented sensitivity and resolution. We demonstrate how information on the fate of nanoparticles can be obtained by retrieving molecular fingerprint information using a combination of hyperspectral Raman spectroscopy mapping, time‐of‐flight secondary ion mass spectrometry (TOF‐SIMS) imaging and density‐dependent color scanning electron microscopy (DDC‐SEM), thus bridging the aforementioned localization gap. The analytical cascade described in this work is directly translatable to most other inorganic nanoparticle systems and shows how biochemically complex nanomaterials can be understood and their interaction with and fate within their host can be comprehended. Such in‐depth characterization and analysis provide additional insights into the material‐biology interplay, which are inaccessible by conventional methods (histological and immunological assays and elemental analysis) and will support the rational development and regulation of a wide range of nanomaterials for biomedical applications.

## 2 Results and Discussion

Flame spray synthesis enables the scalable,^[^ ^32^ ^]^ sterile and well‐controlled synthesis of complex metal oxide nanoparticles with a diversity of compositions and architectures.^[^ ^33^, ^34^ ^]^ Since the stoichiometry of the nanoparticle product made through flame spray pyrolysis corresponds to that of its precursor solution, this technique offers a straightforward way to produce hybrid mixed metal oxide nanoparticles in a tightly controlled scalable process, fulfilling major prerequisites for clinical nanomaterial manufacturing.^[^ ^35^ ^]^ Here, BG/ceria hybrid nanoparticles synthesized by scalable one‐step flame spray pyrolysis were characterized in their as‐prepared state prior to application in vivo. The techniques utilized for the as‐prepared characterization of this complex system are applicable to a wide range of inorganic nanoparticles. While the presented analytical imaging cascade is applicable to any type of nanoparticle administration, the current study focuses on the analysis of topically applied nanoparticles, which is the intended route of administration for these unfunctionalized BG/ceria hybrid nanoparticles.

### 2.1 Characterization of As‐Prepared Nanoparticles

**Figure 1:**
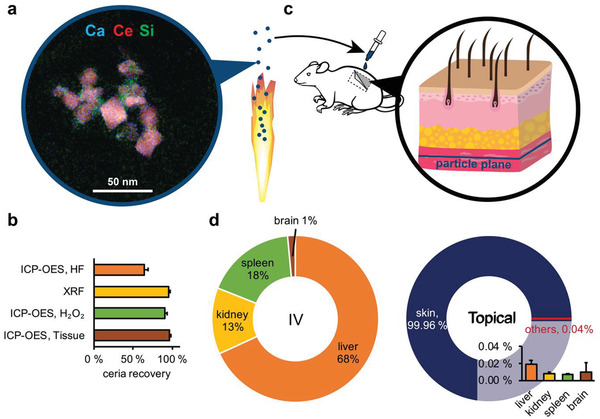
a) BG/ceria hybrid nanoparticles were produced using flame spray pyrolysis. Scanning transmission electron micrograph overlaid with energy‐dispersive x‐ray spectroscopy maps show the elemental distribution within the particles. b) Ceria recovery in the hybrid nanoparticles was quantified using different elemental analysis techniques. The standard digestion method using hydrofluoric acid (HF) fails due to the formation of CeF_3_. Digestion using a mixture of H_2_O_2_ and HNO_3_ showed high recovery rates for both bare particles and on particle‐spiked tissues. c) The nanoparticles in suspension were applied to the subcutis in a rat skin flap model. d) Biodistribution of BG/ceria hybrid nanoparticles 7 days after application (intravenous injection into the inferior vena cava or topical application to the subcutis). In the case of the topical application, more than 99.96% stayed at the initial site of application (*n* = 3).

High‐resolution scanning transmission electron microscopy (STEM) and energy‐dispersive X‐ray spectroscopy (EDXS) show the presence of 20–40 nm crystalline ceria nanoparticles surrounded by amorphous sub‐10 nm BG nanoparticles (**Figure** **1**a; Figure S1, Supporting Information). This phase separation observed in the hybrid nanoparticles is due to the production method which includes two independent precursor inlets, which allow the primary precursor, ceria, to partially oxidize before meeting the secondary precursor (bioglass).^[^ ^25^ ^]^ The obtained morphology of the hybrid oxide nanoparticles is characteristic of flame spray synthesized nanomaterials. X‐ray diffraction (XRD) measurements of the hybrid materials confirm the presence of an amorphous phase as well as a crystalline ceria content of 85.64%, estimated using NiO as an internal standard (Figure S2, Supporting Information). The crystallite size of ceria was estimated by the Scherrer equation as 22 nm and is in good agreement with the electron micrographs. The specific surface area determined by the Brunauer Emmett Teller (BET) method was 43 ± 0.2 m^2^ g^−1^ and the particle size was estimated as *d*_BET_ = 25 nm. Additional physicochemical characterization by FTIR, Raman and XPS of the as‐prepared nanoparticle hybrids can be found in Figure S2 in the Supporting Information. X‐ray photoelectron spectroscopy (XPS) indicates that ceria is present predominantly as Ce^4+^ (Figures S2 and S3, Supporting Information).^[^ ^25^ ^]^ The organic content of the nanoparticle hybrids was determined by thermogravimetric analysis mass spectroscopy (TGA‐MS) and CHN elemental analysis. The total carbon content of as‐prepared nanoparticles was 1.5 wt%, which is in line with carbon contents of similarly produced silica nanoparticles.^[^ ^36^ ^]^ The compositional analysis of the BG/ceria (Ce, Ca, Na, P, Si) particles is analytically challenging, especially because the hydrofluoric acid typically used for the digestion of silica‐based materials forms insoluble CeF_3_ with the cerium present in the sample. If an established digestion method^[^ ^37^ ^]^ for silica‐containing ceria hybrid materials based on a mixture of HNO_3_/H_2_O_2_/HCl/HF was used, only 62% of the theoretical cerium amount was found (Figure 1b). Therefore, the composition of the BG/ceria hybrid has been determined by two complementary methods, wavelength dispersive X‐ray fluorescence (WD‐XRF, not requiring chemical digestion) and inductively coupled plasma optical emission spectrometry (ICP‐OES) using a microwave‐assisted digestion method based on HNO_3_/H_2_O_2_. Elemental analysis of the hybrid nanoparticles by ICP‐OES using an HF‐free digestion route yields 85.87 wt% ceria which is in excellent agreement with the nominal ceria content calculated from the liquid precursors of (86.37 wt%) as well as the content determined by XRD (relative deviation: 0.3 wt%) and WD‐XRF (relative deviation: 4 wt%).

Before in vivo application, the nanoparticles were dispersed in physiological saline. The hydrodynamic diameter in physiological saline was 273 ± 6 nm (dispersity of 0.37) and the zeta potential reached a value of −27 mV at physiological pH (7.3) and −25 mV at lysosomal pH (4.5), in line with previously reported nanoparticle‐based systems with similar composition. Comparable values were found upon contact with other dispersants, with sizes slightly larger and zeta potential values slightly less negative after exposure to human plasma (Table S1, Supporting Information).^[^ ^38^, ^39^ ^]^

### 2.2 In Vivo Application and Biodistribution Measurements by Elemental Analysis

In order to validate the ICP‐OES and ICP‐MS protocols on the tissue samples, they were applied to nanoparticle‐spiked tissue samples to measure the recovery for CeO_2_. For a reverse aqua regia digestion protocol, recovery rates ranged from 92.8% to 103%. For HNO_3_/H_2_O_2_ digestion, recovery rates between 95.2% and 97.9% were found for nanoparticle‐spiked tissue samples. The latter protocol was deemed more robust and used for subsequent digestions. A lower limit of detection was determined (8.3 µg g^−1^ tissue for ICP‐OES and 0.01 µg g^−1^ of tissue for ICP‐MS). Additionally, nanoparticle degradation in lysosomal conditions (pH 4.5) was assessed over a period of 14 days by measuring Ce and Si ion dissolution (Figure S4, Supporting Information). Very low Si release (<2%) from the BG part of the nanoparticles was measured and close to no Ce release (<0.1%). This finding is in good agreement with literature reports showing slow degradation of ceria in intracellular environments^[^ ^40^ ^]^ and indicates that the majority of Si and Ce is present as nanoparticles.

Figure 1c illustrates the topical application of nanoparticle dispersions (0.1 mg of nanoparticles per cm^2^) to the subcutis in rats. Additionally, equivalent nanoparticle doses were applied as bolus intravascular (IV) injection into the inferior vena cava. After seven days, organs were harvested and the cerium concentration in tissue was determined by ICP‐OES and ICP‐MS, using the validated elemental analysis method.

Of the IV injected nanoparticles, 68% accumulated in the liver (Figure 1d) and significant amounts of nanoparticles were also detected in the spleen (18 wt%) and the kidneys (13 wt%). Notably, cerium was also found in the brain, accounting for ≈1 wt% of the applied nanoparticle dose. The crossing of the blood–brain barrier has previously been reported for ultrasmall ceria nanoparticles,^[^ ^41^, ^42^ ^]^ however, the elevated values could also be caused by small amounts of dissolved Ce^3+^ ions. The results from the IV application show a characteristic distribution where most of the nanoparticles are found in the liver, spleen and kidney, which is in agreement with other studies.^[^ ^43^, ^44^ ^]^ Notably, the developed analytical method enables sensitive quantification of these complex metal oxide nanoparticles after in vivo administration. Since the BG/ceria nanoparticles are intended to be used as topical agents, their biodistribution was assessed after topical application using the same protocol. In stark contrast to the IV administration, around 99.96 wt% of the applied nanoparticles were found in the skin tissue at the original site of application (Figure 1d). The remaining 0.04 wt% were found in the liver (0.019 ± 0.005 wt%), the kidney (0.008 ± 0.002 wt%), the spleen (0.007 ± 0.001 wt%), and the brain (0.010 ± 0.011 wt%). Assuming uniform distribution over measured organs, an estimated 104.7% ± 10.6% of the initial dose (2.7 mg) was found in the sampled organs. In brief, after applying the BG/ceria nanoparticles in rats via their intended topical administration route, a large majority of them has stayed where applied while a minimal dose has been distributed to the organs. The elemental analysis study carried out here illustrates how inorganic nanoparticles, even complex ones, can be traced in different organs in a label‐free manner.

### 2.3 Nanoparticle Localization by µCT

**Figure 2:**
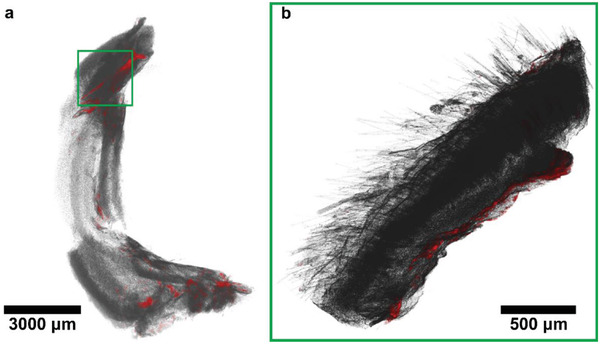
Reconstructed computer tomographs of a) a large part of a rat skin flap and b) a smaller rat skin biopsy. Green frames indicate the same region. High‐intensity pixels are colored red and represent nanoparticles. The nanoparticles are easily identifiable and are located along the subcutis of the rat.

After localizing the bulk of the topically applied nanoparticles at the application site, skin flap biopsies were collected and subjected to high‐resolution microcomputed tomography (µCT) analysis. Agglomerates of high‐density particles can be identified as bright spots in the CT images as seen in **Figure** **2** and Figure S5 (Supporting Information). The nanoparticles are distributed on the subcutis of the rat as a thin layer (≈50 µm) at the initial site of the application.

### 2.4 Identification of the Cellular and Biochemical Environment of the Nanoparticles

**Figure 3:**
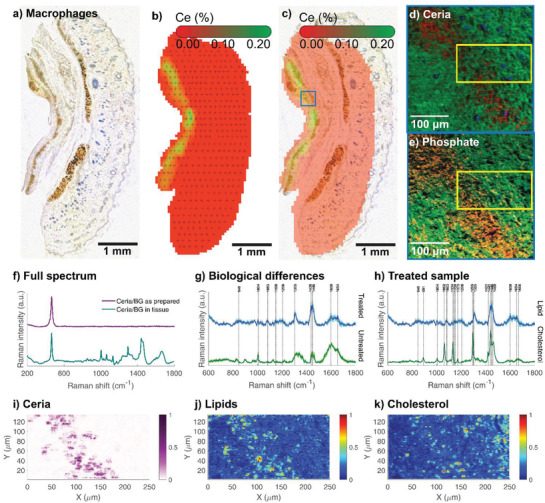
a) Macrophage‐stained (CD68) histology section of skin rat tissue. b) XRF measuring spots and Ce signal. c) Overlay of (a) and (b). The Ce distribution is limited to the subcutaneous region of the skin and is maximal (green) around the macrophages (brown staining). Blue frame indicates ToF‐SIMS measurement region d) ToF‐SIMS map with C*_x_*H*_y_* (green) as a tissue marker, SiO*_x_* (blue) as a substrate marker, and ceria (Ce*_x_*O*_y_*H*_z_*, red). e) The same region with CN + CNO (green), O + OH (blue) and phosphate (PO_2_ + PO_3_, red). The co‐location of ceria and phosphate indicates the mineralization of the BG component. f–k) Raman spectroscopy signatures of tissues from nanoparticle‐treated and untreated rats. Non‐negative matrix factorization (NMF) was used to separate different constituents, compare Figure S8 in the Supporting Information. f) Average Raman spectra of the as‐prepared nanoparticles versus treated tissue. Raman intensity of treated tissue is normalized according to the peak intensity of ceria at 465 cm^−1^. The average spectrum can be decomposed into an inorganic part <600 cm^−1^ and a biological part >600 cm^−1^. g) The lipid component spectra of the treated and untreated tissue samples show a higher occurrence of lipid components in the treated sample. The shaded area denotes the standard deviation from bootstrap resampling. h) The lipid and cholesterol components that were extracted match their specific peaks reported in the literature.^[^ ^59^, ^60^, ^61^ ^]^ i) The ceria component shows the localization of the nanoparticles in the tissue sample. j) There is an increased lipid occurrence around the nanoparticles. k) Additionally, increased amounts of cholesterol are found in the particle region.

After identifying relevant regions by CT, histological sections were cut from skin biopsies and stained with hematoxylin & eosin (H&E), CD68 (macrophages) and CD31 (endothelial cells), respectively (see **Figure** **3**a; Figure S6, Supporting Information). In agreement with previous reports,^[^ ^45^ ^]^ the identification of nanoparticles in histological samples is not straightforward and therefore correlative X‐ray fluorescence mapping was employed to localize regions with high cerium concentrations (Figure 3b,c). XRF mapping of the entire biopsy cross‐section indicates the accumulation of cerium in the ventral part of the skin flap biopsy. Overlay with the CD68 stained tissue section indicates a high number of macrophages in the nanoparticle‐rich tissue region. The two main shortcomings of XRF mapping, namely limited spatial resolution (≈10 µm) and missing information on organic compounds, can be compensated by Raman spectroscopy and time‐of‐flight secondary ion mass spectrometry. ToF‐SIMS analysis of the tissue flap indicates ceria (CeO*_x_* in red, Figure 3d) and phosphate (PO*_x_* in orange, Figure 3e) rich regions surrounded by an organic matrix. Label‐free Raman spectroscopy mapping on histological sections (Figure 3f–k) allows both the identification of particles and organics by their spectral fingerprint.

To further investigate the immediate surroundings of the nanoparticles, hyperspectral image unmixing for the Raman spectroscopic data based on non‐negative matrix factorization (NMF) was performed.^[^ ^46^ ^]^ The abundance map of ceria was constructed by unmixing the Raman spectra in the 200–600 cm^−1^ region where the principal peak of ceria (465 cm^−1^) is present. Similarly, lipids and cholesterol components were extracted by performing NMF in the 600–1800 cm^−1^ biological “fingerprint” region^[^ ^47^ ^]^ where several characteristic peaks of lipids and cholesterol are present. (Figure 3i–k).

Increased levels of lipids and cholesterol were found around the nanoparticles compared to untreated samples and tissue distant from the nanoparticles (Figure 3i–k), with the 2D spatial correlation of ceria and lipid signatures being 60%. This increase of lipid occurrence around BG/ceria nanoparticles is in good agreement with previous in vitro cell culture experiments with BG where it was hypothesized based on gene expression analysis that BG upregulates the mevalonate and sterol biosynthesis pathways.^[^ ^48^ ^]^ ToF‐SIMS based analysis of the organic constituents in the particle‐containing tissue region also showed characteristic signatures of phosphocholines, cholesterol and fatty acids in the proximity of the nanoparticles in support of the Raman findings (Figure S7a–d, Supporting Information). The biomolecular environment and impact of nanomedicines is of high clinical interest. The measurements on the BG/ceria nanoparticles after in vivo application showcase the use of modern spectroscopic techniques to shine light on biological processes in close vicinity of metal oxide nanoparticles (compare Table S2 in the Supporting Information). The accumulation of lipids around the nanoparticles and their biological relevance with regard to angiogenic and tissue regenerative properties of the nanoparticles are yet to be understood.^[^ ^16^ ^]^

### 2.5 Nanoparticle Biotransformation

**Figure 4:**
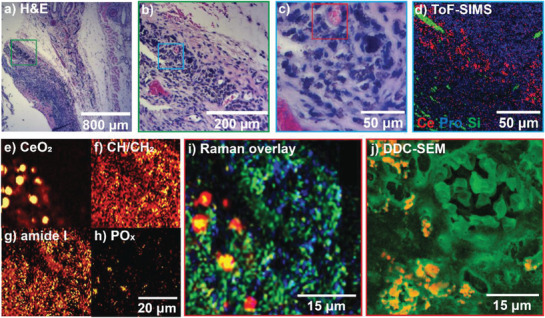
a–c) Histological images of rat skin tissue. Same‐color frames indicate approximate region. d) Corresponding ToF‐SIMS map with proline (blue) as a tissue marker, Si (green) as a substrate marker, and Ce (red). e) Raman merged spectral map of the same region showing CeO_2_, f) CH/CH_2_ (lipids), g) amide I (proteins), and h) PO*_x_* (apatite). i) Overlay of the previous maps with CeO_2_ in red, CH/CH_2_ in blue, amide I in green, and PO*_x_* in blue. j) Electron micrograph of the same region confirms particle distribution around a blood vessel.

Apart from the location and surroundings of therapeutic nanoparticles, their physical and chemical state is highly important to holistically understand their fate. In living organisms, these states are subject to a modification process called biotransformation.^[^ ^49^ ^]^ In the following, we assess the biotransformation of the topically applied BG/ceria nanoparticles. An overlay of the histology with the spectroscopic (XRF, ToF‐SIMS, and Raman) data further indicates that the nanoparticles are found at the initial site of application (Figure 2–**4**). Biological information from H&E stained tissue sections (Figure 4a–c) can be completed by ToF‐SIMS (Figure 4d). ToF‐SIMS showed a characteristic ceria signal from the particles embedded in the surrounding biological matrix (proteins and lipids) only after sputtering away a thin top layer with oxygen. The depth profiling indicates that the ceria is covered by a thin (<5 nm) layer of cerium‐free material. Negative and positive polarity measurements on said layer indicate phosphate (PO^−^, PO^2−^, and PO^3−^) and Ca_3_PO_5_^+^, Ca_4_PO_6_^+^ fragments respectively. These compounds suggest calcium phosphate mineralization of the BG on the particle surface. Higher magnification Raman peak maps of the CeO_2_ F_2g_ signal (465 cm^−1^), CH/CH_2_ vibrations (1448 cm^−1^), and the amide I band (C=O, 1655 cm^−1^) allow localization of ceria, as well as lipid and protein‐rich compounds associated with the particles and the surrounding tissue with an effective resolution of around 500 nm (Figure 4e–g).

The maps give a strong indication that the nanoparticles are present in well‐confined agglomerates with a characteristic length of 10 µm in line with the previous observation of increased macrophage presence in ceria‐rich regions. Additionally, characteristic Raman signatures at 960 cm^−1^ (symmetric stretching of the orthophosphate groups PO_4_^3−^ in apatite) indicate in situ mineralization and formation of apatite on the particles (Figure 4f). As seen on a Raman peak map overlay, this mineralization coincides well with the ceria distribution (Figure 4i) and is in line with the ToF‐SIMS results. No formation of CePO_4_ was found on the surface (no peak at 973 cm^−1^, stretching vibrations of the ‐PO_4_ unit of monoclinic CePO_4_). This absence of CePO_4_ is in good agreement with previous studies using X‐ray absorption spectroscopy showing Ce reduction and CePO*_x_* formation primarily for small Ce nanoparticles and almost no reduction for ceria particles with a size of 20–30 nm.^[^ ^50^ ^]^ The formation of apatite upon exposure to body fluids is in line with previous in vitro studies, which report the detection of apatite on BG/ceria hybrid nanoparticles following exposure to simulated body fluid.^[^ ^25^ ^]^ The formation of apatite on BG particles is also well described following in vivo application.^[^ ^51^ ^]^

### 2.6 Electron Microscopy Observations on Nanoparticles in Tissue

**Figure 5:**
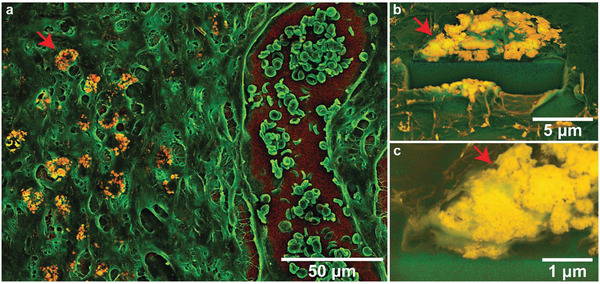
a) Density‐dependent color secondary electron micrograph of nanoparticle‐containing tissue macrophages next to a blood vessel. Nanoparticles show up in yellow (compare EDXS in Figure S8 in the Supporting Information). b) Focused‐ion beam cut trench giving access to cross‐sectional imaging of a c) nanoparticle‐containing macrophage. Red arrows indicate the same region.

Since the resolution of the Raman spectroscopy maps is diffraction‐limited (the effective lateral resolution is around 500 nm), tissue sections were also analyzed by electron microscopy (Figure 4j and **5**). Backscattering electron (BSE) micrographs allow straightforward identification of the electron‐dense, ceria‐containing nanoparticles in the tissue samples as confirmed by EDXS (Figure S9, Supporting Information). Secondary electron (SE) images and backscattering electron (BSE) images were collected from the same region in order to gather information on both the tissue morphology and the elemental contrast. These images were then assembled into RGB stacks; the SE image was assigned to the green channel and the BSE data was colored in red (Figure S10, Supporting Information). Overview density‐dependent color scanning electron micrographs images were overlaid with corresponding histological micrographs and allow straightforward identification of particle‐rich regions on the entire histology (Figure S11, Supporting Information). These DDC‐SEM micrographs at different magnifications again strongly suggest that nanoparticles remain localized at the primary site of application, i.e., the ventral part of the skin flap. DDC‐SEM imaging at higher magnification allows clear identification of accumulated nanoparticles in tissue sections based on their density and structural characterization at nanometric resolution (Figure 5c). In contrast to TEM analysis, DDC‐SEM allows analysis of a much wider field of view and provides a powerful tool for the straightforward identification of nanoparticle‐rich regions in histological sections. Overlaying these with DDC‐micrographs with CD68‐stained sections confirms the co‐localization of the particle agglomerates and CD68‐positive macrophages (Figure S9c,d, Supporting Information). All nanoparticles are densely packed in the macrophages, whereas almost none are found in the connective tissue. To gain more insights into the particle localization, a trench was cut by focused ion beam milling confirming that the nanoparticles are indeed localized within membrane‐bound structures (Figure 5b,c). The chemical composition of the nanoparticles was again confirmed by EDXS, which shows emissions characteristic for cerium, phosphorus, and silicon (Figure S9, Supporting Information). This finding confirms the hypothesis that macrophages take up most nanoparticles and shows how they are densely packed within them. The co‐localization of nanoparticles with macrophages has been previously observed for metal and metal oxides.^[^ ^43^, ^44^, ^45^, ^46^, ^47^, ^48^, ^49^, ^50^, ^51^, ^52^ ^]^

The proposed imaging and analysis cascade enabled the tracing of topically applied inorganic nanoparticles from organ biopsies (10^−2^ m) to subcellular structures (10^−8^ m). While this in‐depth analysis confirms the presence of cerium, silicon, and phosphorus at the initial site of application, the current study also illustrates that crystalline, high‐*z*, poorly soluble components (such as ceria) are easier to trace compared to amorphous, light and potentially degradable constituents (e.g., BG). This cascade is transferable to a wide range of metal and metal oxide nanoparticles (see Table S2 in the Supporting Information).

## 3 Conclusions

In this work, we have demonstrated the integration of cutting‐edge imaging and analysis techniques to trace the location and alteration of nanoparticles in tissue following topical application. The cascade provides insights into the nanoparticle intratissural distribution which is inaccessible by solely conventionally used techniques, such as histology and elemental analysis. The studied nanoparticles showed very low clearance and stayed at the site of application for more than a week. However, there are clear physicochemical changes measurable, showing bioactivity and biotransformation of the investigated particles. Notably, the proposed analytical cascade capitalizes on label‐free methods and thus no modification to the active agents is needed. The inorganic nanoparticles and their degradation products, as well as alternations in the surrounding tissue, can be assessed directly instead of relying on surrogate markers. Additionally, the multiscale correlation of different imaging and analysis techniques proposed here is applicable to a wide range of inorganic nanomedical agents due to the versatile nature of the used techniques. In the face of an ever‐growing number of inorganic nanomedicinal products (including more exotic materials such as HfO_2_) on their way to clinics, this cascade can provide a basis for both the improvement of their effectiveness and their regulation. We believe that the demonstrated multiscale assessment of nanoparticle‐tissue and cell interactions will facilitate the rationalized and sustainable design and regulation of new nanomaterials.

## 4 Experimental Section

##### Nanoparticle Synthesis

Chemicals were purchased from Sigma Aldrich if not stated otherwise. BG/ceria hybrid nanoparticles were produced by liquid‐feed flame spray pyrolysis according to previously published methods.^[^ ^25^ ^]^ The particles were produced in a two‐nozzle setup (see Matter et al.,^[^ ^25^ ^]^ Figure S1, Supporting Information): Bioglass was used as secondary precursor and ceria as primary precursor. 6 mL min^−1^ of primary precursor (total metal ion concentration 0.3 m in THF; 40 wt% calcium acetylacetonate hydrate, 37 wt% sodium 2‐ethylhexanoate, 6 wt% tributyl phosphate, 17 wt% HMDSO) was injected into a water‐cooled spray nozzle and dispersed by 5 L min^−1^ O_2_. The pressure drop at the nozzle tip was approx. 1.5 bar. The spray was then ignited by a premixed CH_4_:O_2_ (1.25 L min^−1^:2.5 L min^−1^) flamelet. The entire flame and spray were enclosed in a stainless steel tube and additionally sheathed with 20 L min^−1^ O_2_. In addition, 11 cm down the particles stream and at an angle of 45°, the secondary precursor (0.3 m Cerium(III) 2‐ethylhexanoate in THF) was fed at 3 mL min^−1^ through a second nozzle and dispersed with N_2_ (13 mL min^−1^). The as‐prepared nanoparticles were collected from a glass fiber filter mounted above the flame. Nanoparticle suspensions were prepared by tip‐sonication immediately before application.

##### Phyiscochemical Characterization

Scanning transmission electron micrographs and elemental distribution maps of the as‐prepared nanoparticles were recorded on a Talos F200X TEM (Super‐X EDS, 4 detector configuration, FEI, USA) at an accelerating voltage of 200 kV. Samples were mounted on a doubletilt holder and fixed using a molybdenum ring and clamp. The data were processed using the software Velox 2.9 (FEI, USA). X‐ray diffraction patterns (XRD) were obtained with a Bruker D8 advance diffractometer (40 kV, 40 mA, CuKa radiation) at 2*q* = 10–70°. Dynamic light scattering (DLS) hydrodynamic size and zeta potential measurements were conducted using a Zetasizer (Nano ZS90, Malvern Instruments). To assess the effect of different fluid compositions, nanoparticles were suspended in water (ddH_2_O), PBS, citrate buffer or human plasma and incubated for 1 h. Nanoparticles were subsequently centrifuged and resuspended in 10% PBS for zeta potential measurements (Table S1, Supporting Information). Raman spectra of the nanoparticles were recorded on a WITec alpha 300R confocal Raman microscope, equipped with a UHTS 300 Vis spectrometer and an Andor Newton EMCCD (see below). Fourier transform infrared spectra were collected using a Varian 640‐IR spectrometer. For X‐ray photoelectron spectroscopy (XPS), the as‐prepared nanoparticles were analyzed on a PHI 5000 VersaProbe II instrument (USA) with a monochromatic AlK*α* X‐ray source. Energy resolution was at 0.8 eV per step at a pass‐energy of 187.85 eV for survey scans and 0.125 eV per step and 29.35 eV pass‐energy for high‐resolution region scan of the Ce3d5 region, respectively. Carbon C1s at 284.5 eV was used as a calibration reference. Data analysis was performed with CasaXP software (Casa Software Ltd, United Kingdom). Peak fitting was done by adaptations to Sims et al.^[^ ^53^ ^]^ and Bêche et al.^[^ ^54^ ^]^

##### Animal Model and Tissue Procurement

The experimental animal study was approved by the Ethics Committee for Animal Experimentation, Bern, Switzerland (Approval number 89/16). The experimental section of the animal model has been published before.^[^ ^16^ ^]^ Briefly, A 9 cm × 3 cm dorsal perforator‐based flap was raised in Lewis rats and the interface was treated with the nanoparticle suspension (in saline at a concentration of 2.7 mg mL^−1^, dose per area: 0.1 mg cm^−2^, total dose per animal: 2.7 mg) in an attempt to increase flap survival. The skin was closed with a running suture and the flap was removed again for analysis seven days after treatment. Various biopsies were harvested and fixed in formalin, while the remaining of the flap was frozen in liquid nitrogen. For the systemic administration, the nanoparticle suspension (total dose per animal: 2.7 mg) was applied intravenously into the inferior vena cava. Tissues were again harvested and frozen in liquid nitrogen or fixed in formalin. In addition to liver, kidney, spleen, lung, brain and skin tissue also blood samples were collected for further analyses.

##### Elemental Analysis

Carbon content of the nanoparticles was measured by using a carbon and sulfur analyzer (LECO CS‐844). The nanoparticles were burned at approximately 2000 °C and carbon was detected as CO_2_ using an infrared cell.

Wavelength Dispersive X‐ray Fluorescence (WDXRF) was used (Rigaku Corporation; Tokyo, Japan) to measure Ce‐content both directly on the nanoparticle powder and a pressed pill. Ce‐content in the different rat organs was measured by taking organ samples of around 0.05–0.35 g. The samples were then transferred to a PTFE container and mixed with 6 mL 65% HNO_3_ p. a. (Merck) and 1 mL 30% H_2_O_2_ p.a (Merck). The samples were then digested in a microwave (MLS Start) and first measured on an ICP‐OES (Agilent 5110) and then on a QQQ‐ICP‐MS (Agilent 8800).

##### Particle Degradation in Lysosomal Buffer

The particles were suspended in potassium citrate buffer at pH 4.5 to mimic lysosomal pH.^[^ ^56^ ^]^ A concentration of 50 µg mL^−1^ was used. The stock suspension was split in tubes and left on a shaker at 37 °C. The tubes were collected at different days and centrifuged at 20 000 × *g* for 30 min. Diluted samples were analyzed by ICP‐MS.

##### MicroCT

For microCT measurements, the tissue sample was mounted in a polystyrene tube and kept in saturated ethanol atmosphere during the measurement. X‐ray microCT was performed using an EasyTom XL Ultra 230‐160 micro/nano‐CT scanner (RxSolutions SAS, Chavanod, France). The scans were performed using a Varian PaxScan 2520DX detector. The tube was operated at 70 kV and 300 µA current for the large skin flap sample and at 50 µA for the small biopsy. The voxel sizes of the CT scans were 22.5 µm for the large sample and 3 µm for the small biopsy, respectively. The images were acquired at 2 frames per second speed and averaging 10 frames per projection.

##### Histology

Fixed skin biopsies were embedded in paraffin and cut into sections. Selected sections were stained with hematoxylin‐eosin. Macrophages were identified by detection of the CD68 protein by immunohistochemical staining (Sophisto Lab, Muttenz, Switzerland). Light microscopy images of the sections were recorded using a Zeiss Primovert microscope (Zeiss, Feldbach, Switzerland).

##### XRF Tissue Maps

X‐ray fluorescence (XRF, Fischerscope X‐RAY XDV‐SDD, Fischer Technology) has been used to estimate Ce at% along the rat skin tissue. Histological sections mounted on (ITO‐coated) glass slides were deparaffinised and analysed. Mapping measurements were performed at different points covering all the sample area. Concentration maps were created by interpolation using TRIPACK.^[^ ^57^ ^]^

##### TOF‐SIMS

ToF‐SIMS analysis (TOF.SIMS.5 instrument from ION‐TOF GmbH) was performed to investigate the lateral distribution of the BG/ceria nanoparticles within the tissue. Acetone‐fixed histological sections mounted on (ITO‐coated) glass slides were analysed. The data were acquired in imaging mode to provide high lateral resolution (≈200 nm) with 50 keV Bi_3_^++^ primary ions (0.03 pA). An electron flood gun was used to compensate for the charge accumulation in the insulating tissue material. Secondary ions of positive and negative polarities were analyzed from mass 1 to 800 mass units, and from surface areas of 500 × 500 µm^2^ with a resolution of 256 × 256 pixels^2^. In order to increase the ceria signal intensity, the bioglass was gently ablated by using 1 keV O_2_ sputtering for 1 second per scan (positive polarity only). A total of 1300 (400) scans were acquired for positive (resp. negative) polarities to insure a reasonable signal over noise ratio.

##### Raman Microscopy

Raman measurements were performed as described elsewhere.^[^ ^58^ ^]^ Measurements were done either directly on nanoparticles or histological sections that had been deparaffinized with xylene. Measurements were performed on a WITec alpha 300R confocal Raman microscope, equipped with a UHTS 300 Vis spectrometer and an Andor Newton EMCCD. A linearly polarized 532 nm laser was used for excitation. A 50× long distance objective was used (Zeiss, 50×/0.55 NA). Single spectra were acquired with an integration time of 2 s and in at least 5 different locations. A laser intensity of 8 mW and integration times of 2s were used

##### Raman Image Unmixing

Raman spectra were pre‐processed by carrying out cosmic ray removal and baseline subtraction. The number of components for running NMF was set to the number of significant principal components (PCs); the latter was estimated by self‐referencing where the PC correlation coefficients of two random subsamples were used (Figure S8, Supporting Information). NMF was then performed to construct the component spectra and corresponding abundance maps from regions 200–600 cm^−1^ for ceria and 600–1800 cm^−1^ for lipids and cholesterol. All computations were run on MATLAB R2018a.

##### Electron Microscopy

For secondary electron imaging, paraffin embedded samples were dewaxed by immersion in pure xylene for two ten minute intervals, dehydrated through a graded ethanol series (20, 30, 40, 50, 60, 70, 80, 90, and 100% (v/v)) and air dried. The slides were then silver painted, carbon coated and secured on an SEM silver stub using conductive carbon adhesive tape. A Hitachi S‐3499N SEM was used at a voltage of 10 kV for acquisition of backscattered (BSE) and secondary (SE) electron micrographs. The density dependent colour SEM (DDC‐SEM) micrographs were produced by the superimposition of the BSE and SE images using the Adobe Photoshop CC software.^[^ ^59^ ^]^

The focused ion beam (FIB) scanning electron micrographs on deparaffinized histology sections (coated with 10 nm C using Leica sputter coater) were acquired using FEI Helios 660 G3 UC FIB/SEM (10 kV, 0.4 nA) equipped with through‐lens (TLD) backscattering detector. The trenches of 15 µm wide and 10 µm deep were cut by operating the focused gallium ion beam at 30 kV and varying the beam currents between 47 nA to 0.79 nA. The subsequent images were acquired using a TLD detector using 10 kV acceleration voltage and 0.4 nA electron beam current.

## Conflict of Interest

The authors declare no conflict of interest.

## Supporting information

Supporting Information
