# Supplementary material for: Multiscale Analysis of Metal Oxide Nanoparticles in Tissue: Insights into Biodistribution and Biotransformation
Source: Adv Sci (Weinh). 2020 Jun 18;7(15):2000912. doi: 10.1002/advs.202000912 (PMC7404155; doi:10.1002/advs.202000912)
Supplement: Supplementary file 1 — Supporting Information [file ADVS-7-2000912-s001.pdf]

© 2020 Wiley-VCH GmbH

ADVANCED SCIENCE

Open Access

Supporting Information

for *Adv. Sci.,* DOI: 10.1002/advs.202000912

Multi-scale Analysis of Metal Oxide Nanoparticles in Tissue: In-sights into Biodistribu-tion and Biotransformation

*Martin T. Matter, Jian-Hao Li, Ioana Lese, Claudia Schreiner, Laetitia Bernard, Olivier Scholder, Jasmin Hubeli, Kerda Keevend, Elena Tsolaki, Enrico Bertero, Sergio Bertazzo, Rob-ert Zboray, Radu Olariu, Mihai Constantinescu, Renato Figi, Inge K. Herrmann**

Copyright WILEY-VCH Verlag GmbH & Co. KGaA, 69469 Weinheim, Germany, 2018.

Supporting Information

Multi-scale Analysis of Metal Oxide Nanoparticles in Tissue: In-sights into Biodistribution and Biotransformation

*Martin T. Matter, Jian-Hao Li, Ioana Lese, Claudia Schreiner, Laetitia Bernard, Olivier Scholder, Jasmin Hubeli, Kerda Keevend, Elena Tsolaki, Enrico Bertero, Sergio Bertazzo, Robert Zboray, Radu Olariu, Mihai Constantinescu, Renato Figi, Inge K. Herrmann**

**ingeh@ethz.ch, inge.herrmann@empa.ch, +41 (0)58 765 7153*

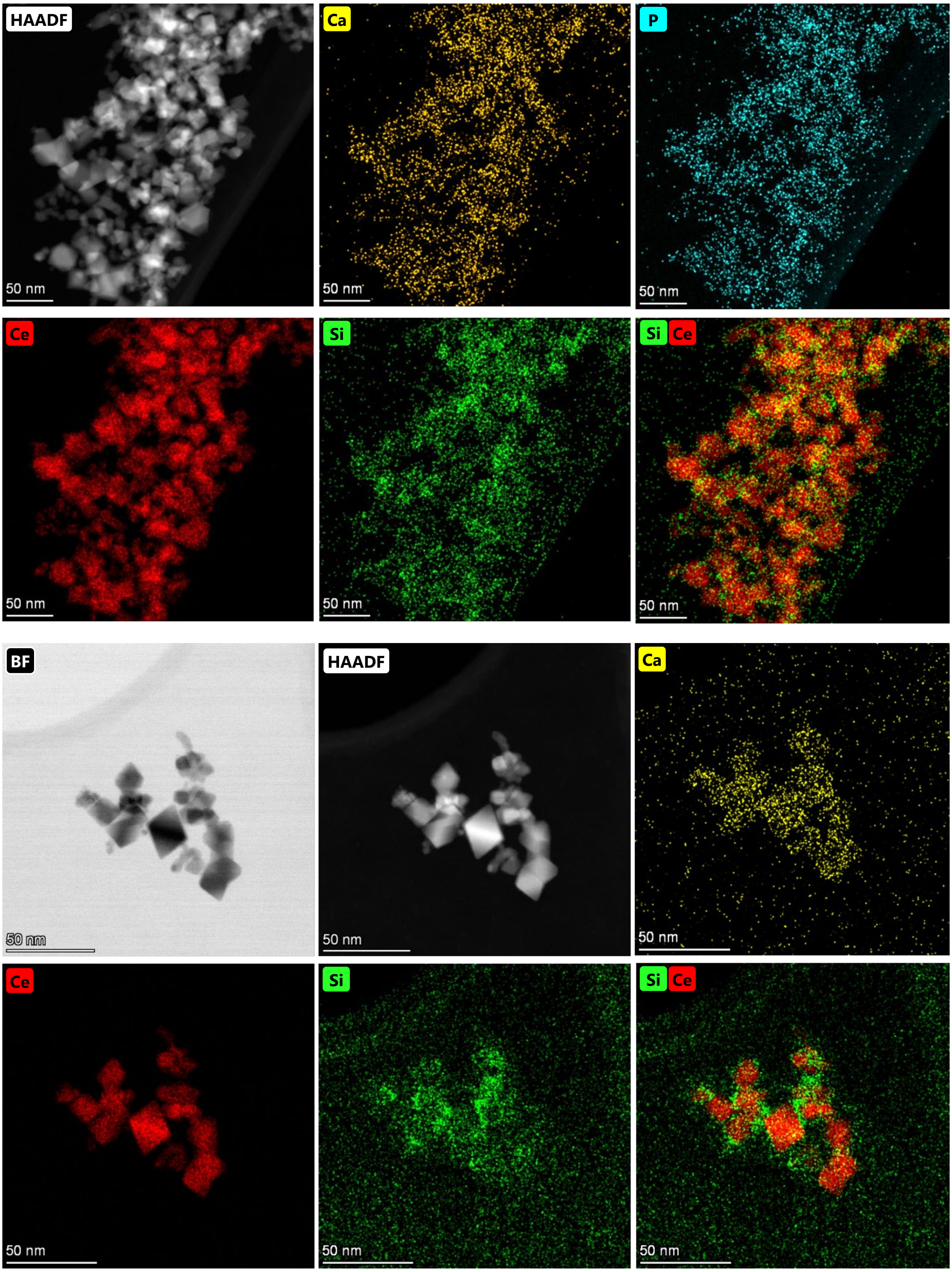

Figure S1: *Bright field (BF) and high-angle annular dark-field (HAADF) scanning transmission electron micrographs of the as-prepared nanoparticles. Dispersive X-ray spectral maps were recorded in two regions of the sample. Peak maps for different elements are shown. The maps indicate a co-localization of ceria and bioglass. Ceria appears as larger crystals, whereas bioglass is diffusely located around the ceria crystals.*

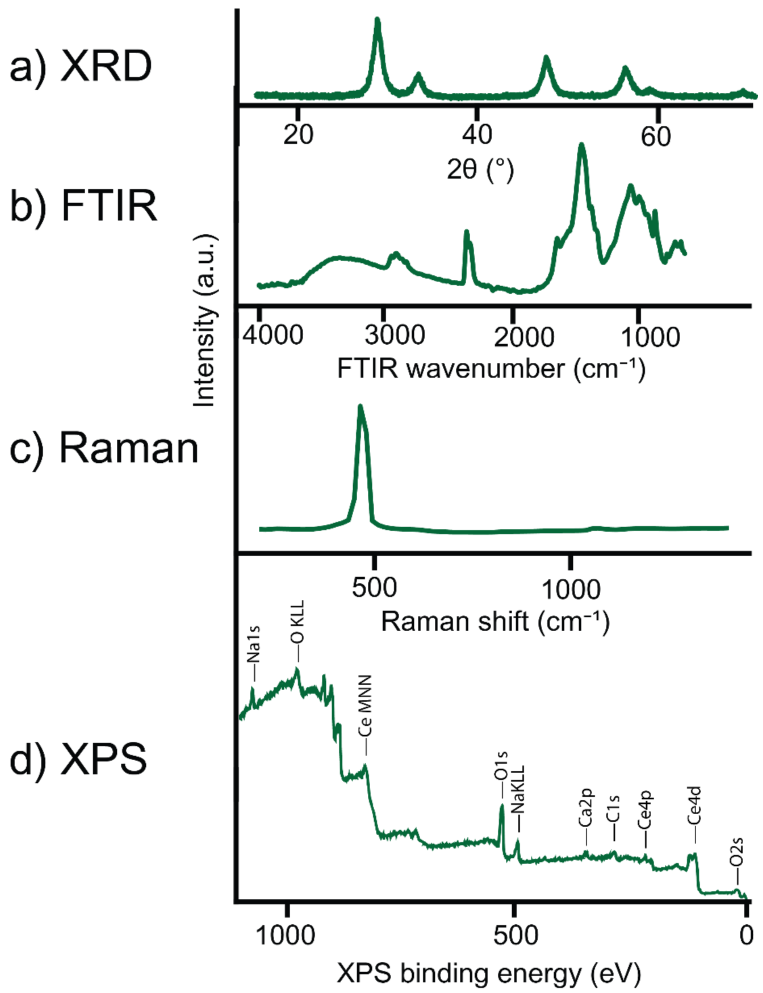

Figure S2: *a) XRD pattern showing a characteristic ceria peaks*[1]*. b) FTIR spectrum showing no indications for carboxylic acid (C=O stretch around 1710 cm^{-1}) groups on the nanoparticles. Additionally, it is missing the hydroxyapatite peak appearing at around 960 cm^{-1}, indicating non-crystalline bioglass in the as-prepared state. c) Raman spectroscopy only shows a pronounced peak at 465 cm^{-1} which is characteristic for ceria. d) XPS survey scan of the as prepared nanoparticles reveals the presence of Ce and bioglass components (Na, Ca).*

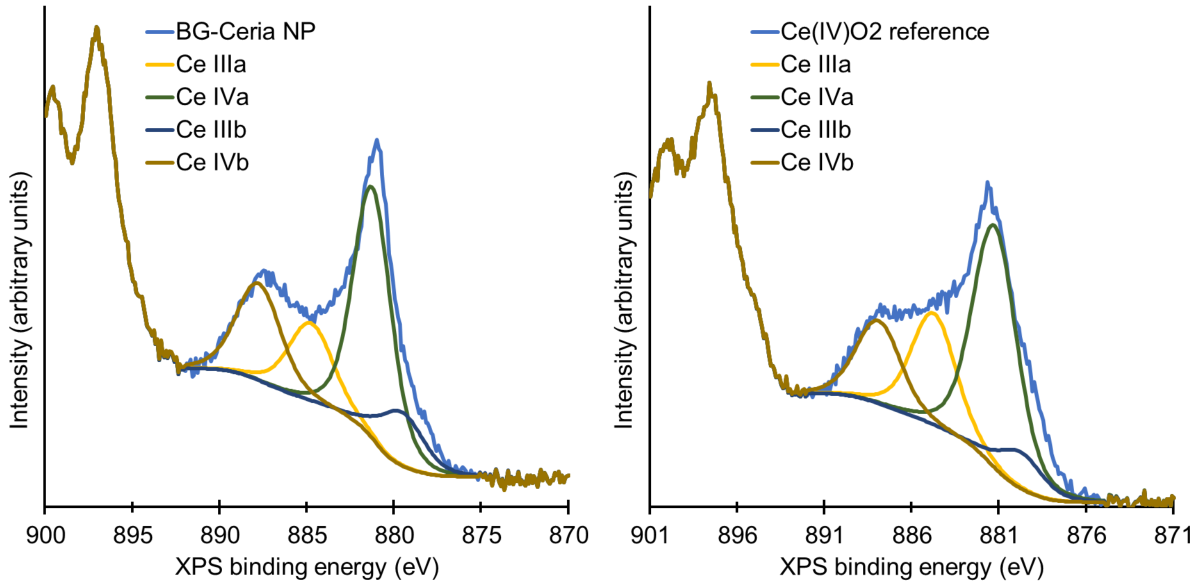

BG/ceria NP	Position (eV)	Concentration (at%)
Ce IIIa	885.0	23.6
Ce IVa	881.5	51.2
Ce IIIb	879.8	7.7
Ce IVb	888.0	17.5

Ce(IV)O_2 reference	Position (eV)	Concentration (at%)
Ce IIIa	885.0	17.6
Ce IVa	881.5	48.4
Ce IIIb	880.0	12.1
Ce IVb	888.1	21.9

Figure S3: *Fittings of the Ce3d5 regions to estimate the Ce(III) and Ce(IV) surface content. Ce(IV)O_2 nanopowder from Sigma-Aldrich was used as a reference. Quantitative results of the two particles are shown in the tables. The as-prepared nanoparticles are rich in Ce(IV).*

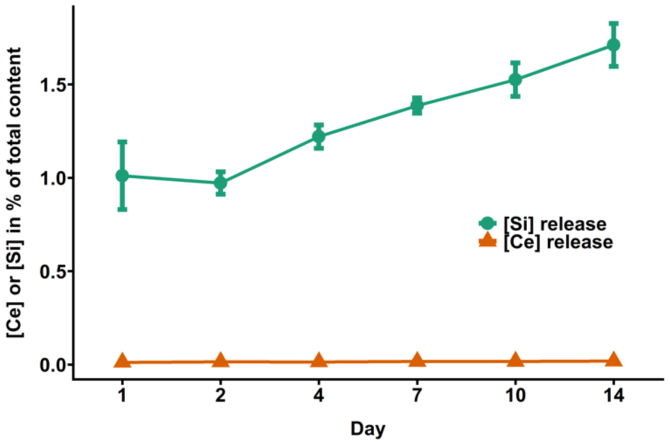

Figure S4: *Si and Ce ion release as a function of total content in nanoparticles. Particles were incubated in lysosomal buffer (pH 4.5) for up to 14 days. As reference, the animal study was conducted for 7 days.*

Table S1: Dynamic light scattering results of the BG/ceria nanoparticles upon suspension in different dispersants, subsequent centrifugation and resuspension in 10% PBS.

	hydrodynamic size (nm)	zeta potential (mV)
ddH_2O	299±20	-26±2
PBS	322±17	-21±1
Citrate buffer	303±3	-37±2
Human plasma	727±66	-15±1

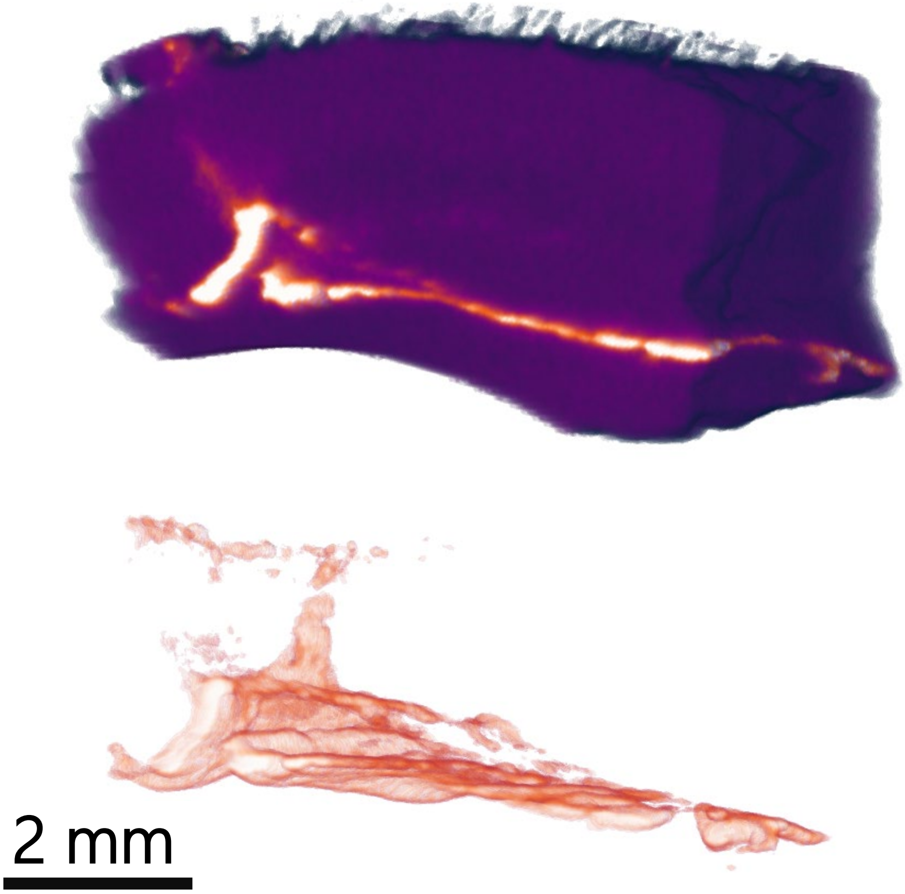

Figure S5: *Reconstructed computer tomographs of a skin flap. High intensity pixels are represented by red and white colors and represent nanoparticle agglomerates. The nanoparticles are easily identifiable and are located along the subcutis of the rat and along a blood vessel.*

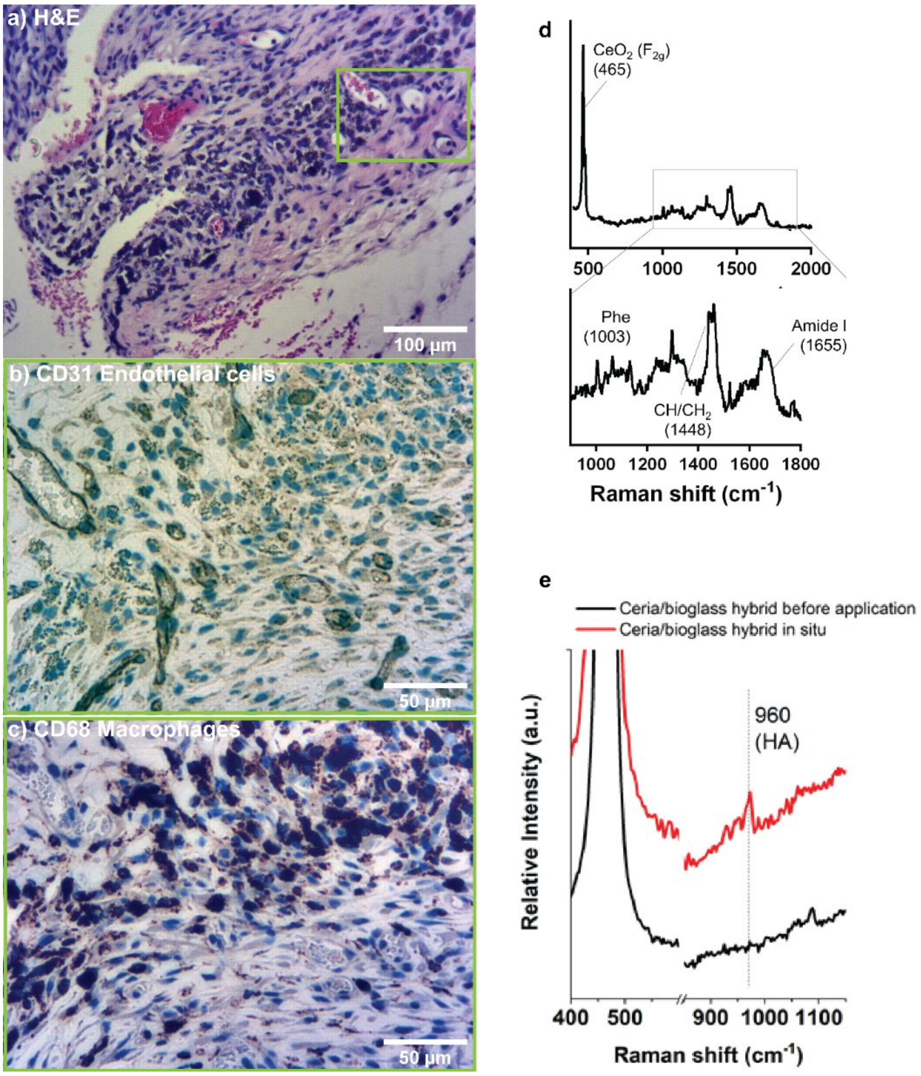

Figure S6: *(a) H&E stained rat skin tissue section. Green frame indicates same region (b) CD31 stained section shows endothelial cells in dark brown. (c) Corresponding region stained for CD68 with activated macrophages in black. d) Raman spectra of the measured region. The zoom-in at the bottom shows characteristic biological peaks. e) Raman spectroscopy shows the formation of hydroxyapatite on the nanoparticle surface after contact with biological fluid. The peak at 960 cm*$^{-1}$ *indicates the mineralization of bioglass.*

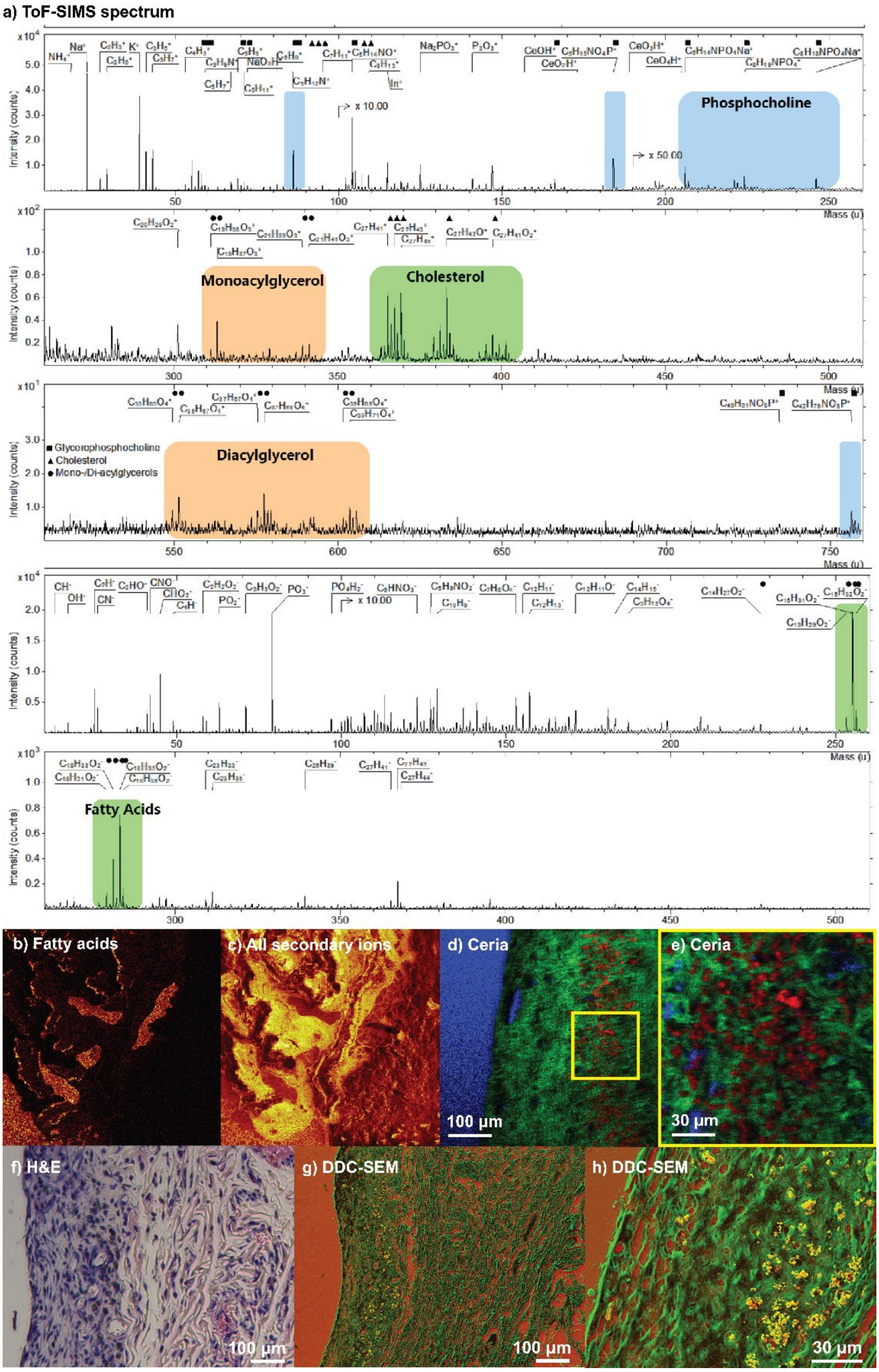

Figure S7: *(a) ToF-SIMS spectrum of the nanoparticle-treated tissue. (b, c) Peak intensity maps of the same region on a nanoparticle-treated skin tissue section. There is a high occurrence of lipids. (d ,e) ToF-SIMS element distribution show ceria (red) on the tissue (green). (f) To (d) corresponding histological section (H&E stained). (g, h) Density-colored scanning electron micrographs of the corresponding region showing nanoparticles in yellow and tissue in green.*

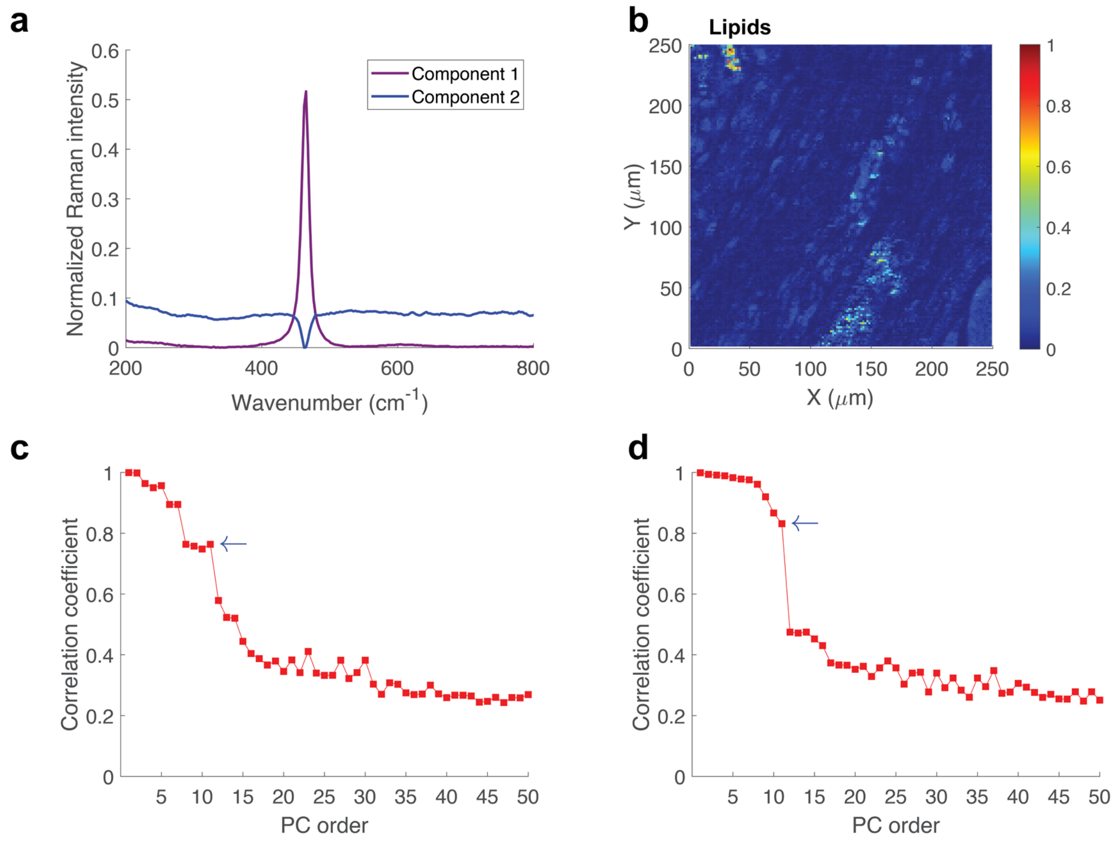

Figure S8*: Raman spectroscopy signatures of nanoparticle-treated tissue. Non-negative matrix factorization (NMF) was used to separate different constituents, compare Figure 3. (a) Ceria (component 1) in the treated tissue can be readily extracted by performing NMF with 2 components in the 200-600 cm^{-1} region (ceria abundance map shown in Fig. 3i). (b) Lipid abundance map of untreated tissue. There is no increased lipid content as opposed to the treated sample. Largest correlation coefficients generated at each PC order (in descending variance) from running self-referencing 1000 times for untreated (c) and treated (d) samples. In each run of self-referencing, the sample was divided randomly into two equal-sized subsamples and their PC correlation coefficient matrix was constructed. The correlation coefficient of, say, PC order 3 was set to the maximum absolute value in row & column 3 of the matrix. PCs resulting from (high-frequency) noises[2][3] can be effectively identified which produce low subsample PC correlations. The number of significant PCs for treated and untreated samples were both determined to be 11 (blue arrows), where the largest decrease of correlation coefficient occurs.*

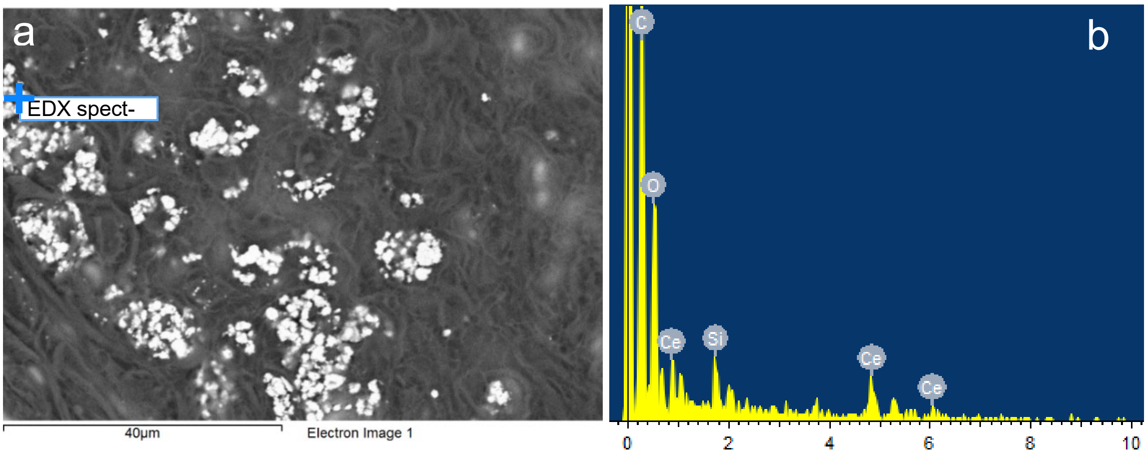

Figure S9: *(a) Backscattered electron microgram showing high contrast for the nanoparticles. An energy dispersive X-ray spectrum was measured at the location indicated with a blue cross. (b) The energy dispersive X-ray spectrum shows strong signals for cerium (Ce) and silicon (Si, the main component of bioglass), indicating the presence of BG/ceria hybrid nanoparticles.*

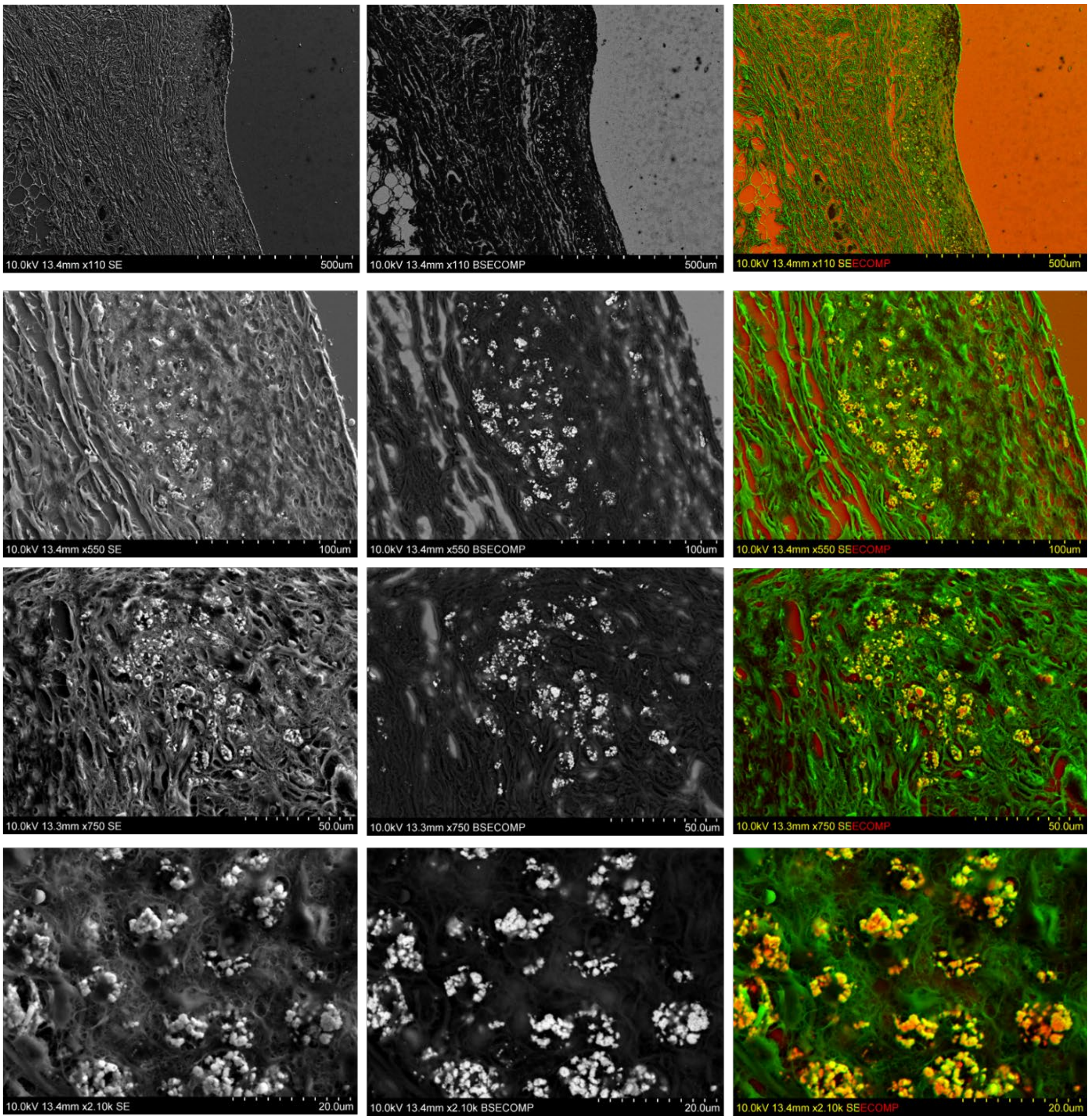

Figure S10: *The first column are secondary electron (SE) micrographs that show the topography of the skin tissue section. The second column are backscattered electron (BSE) micrographs that show the particles as bright clusters, due to their high mass compared to the biological tissue around them. The third column shows an RGB overlay of the first two columns that allows for the observation of both the tissue and the nanoparticles with high contrast. The SE images have been assigned to the green, the BSE images to the red channel.*

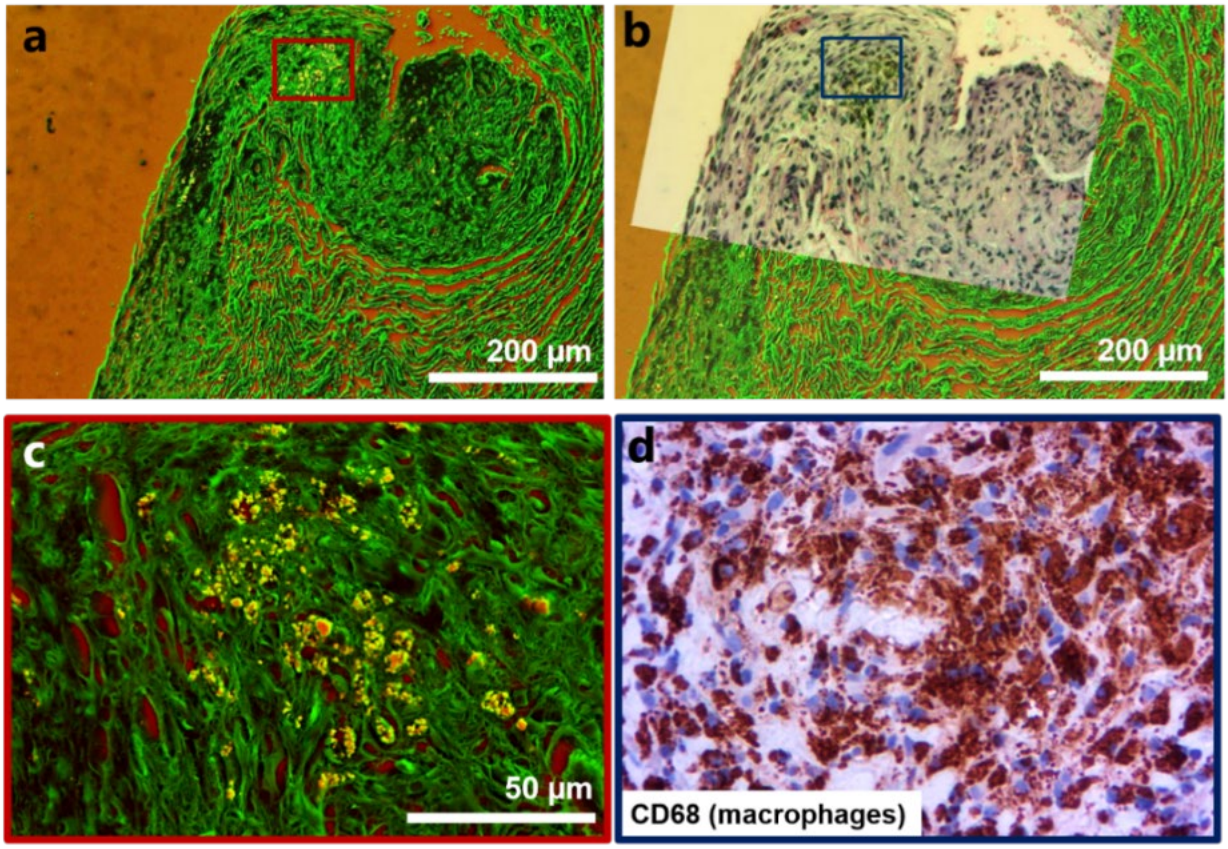

Figure S11: *(a) Density-colored scanning electron micrographs of a skin section showing nanoparticles in yellow and tissue in green. (b) Overlay with corresponding region in histological section. (c) Higher magnification electron micrograph of the nanoparticles in tissue. (d) Corresponding region in histological section (CD68 stained, macrophages in brown). Particle localization correlates with macrophage distribution. Red and blue frames indicate same region.*

Table S2: Applicability of the presented analytical cascade for nanoparticle analysis in tissue.

	(LA, sp-) ICP	CT	Histology	XRF	ToF-SIMS	Raman	EM
Resolution	cm-µm	mm-µm	µm	µm	sub-µm	sub-µm	nm
Sensitivity	ppb (sp)	‰	‰	ppm	ppm	≈sp	sp
Complexity/costs	medium	medium	low	medium	high	low	medium
Metal / Metal ox. NPs	++[4]	++[5]	+	++[6]	++[7]	+[8][9]	++
• Iron-based	+[10]	++[11]	+[12]	++[13]	++	++[14][15]	++[16]
• Cerium-based	++[17][18]	++[19]	+[20]	++[17][21][22]	++[22]	++[23]	++
• Au-based	++[24]	++[27]	+[28]	++[29]	++	++	++[32]
• Si-based	+[25](*[26])	-	-	+[30]	+[31]	-	+[33]
Polymer-based NPs	(*)	--	--	(*)	++[34]	++[35]	-
Carbon-based NPs	(*)	--	--	(*)	+	++[36]	-
Lipid-based NPs	(*)	--	--	(*)	*[37]	*[38]	-

++ very straightforward, + straightforward, - challenging, -- not feasible, * doable with labels, ** straightforward with labels, sp: single particle

References

[1] M.F. Bekheet, M. Grünbacher, L. Schlicker, A. Gili, A. Doran, J.D. Epping, A. Gurlo, B. Klötzer, S. Penner, *CrystEngComm* **2018**, *21*, 145.

[2] M. Wang, W. Wang, L. Shao, *Chin. J. Chem. Phys.* **2018**, *31*, 818.

[3] H. Shen, Y. Liang, O.M. Kvalheim, R. Manne, *Chemom. Intell. Lab. Syst.* **2000**, *51*, 49.

[4] Y.-S.S. Yang, P.U. Atukorale, K.D. Moynihan, A. Bekdemir, K. Rakhra, L. Tang, F. Stellacci, D.J. Irvine, *Nat. Commun.* **2017**, *8*, 14069.

[5] D. Kim, J. Kim, Y.I. Park, N. Lee, T. Hyeon, *ACS Cent. Sci.* **2018**, *4*, 324.

[6] M.J. Pushie, I.J. Pickering, M. Korbas, M.J. Hackett, G.N. George, *Chem. Rev.* **2014**, *114*, 8499.

[7] B. Hagenhoff, D. Breitenstein, E. Tallarek, R. Möllers, E. Niehuis, M. Sperber, B. Goricnik, J. Wegener, *Surf. Interface Anal.* **2013**, *45*, 315.

[8] J. Moger, B.D. Johnston, C.R. Tyler, *Opt. Express* **2008**, *16*, 3408.

[9] R.M. Goodhead, J. Moger, T.S. Galloway, C.R. Tyler, *Nanotoxicology* **2015**, *9*, 928.

[10] B. Chertok, A.J. Cole, A.E. David, V.C. Yang, *Mol. Pharm.* **2010**, *7*, 375.

[11] E. Alphandéry, *RSC Adv* **2019**, *9*, 40577.

[12] K. Tsuchiya, N. Nitta, A. Sonoda, A. Nitta-Seko, S. Ohta, H. Otani, M. Takahashi, K. Murata, K. Murase, S. Nohara, K. Mukaisho, *Int. J. Nanomedicine* **2011**, *6*, 1587.

[13] K. Matusiak, A. Skoczen, Z. Setkowicz, A. Kubala-Kukus, I. Stabrawa, M. Ciarach, K. Janeczko, A. Jung, J. Chwiej, *Nanotoxicology* **2017**, *11*, 1225.

[14] L. Ahlinder, B. Ekstrand-Hammarström, P. Geladi, L. Österlund, *Biophys. J.* **2013**, *105*, 310.

[15] M. Testa-Anta, M.A. Ramos-Docampo, M. Comesaña-Hermo, B. Rivas-Murias, V. Salgueiriño, *Nanoscale Adv* **2019**, *1*, 2086.

[16] M. Levy, N. Luciani, D. Alloyeau, D. Elgrabli, V. Deveaux, C. Pechoux, S. Chat, G. Wang, N. Vats, F. Gendron, C. Factor, S. Lotersztajn, A. Luciani, C. Wilhelm, F. Gazeau, *Biomaterials* **2011**, *32*, 3988.

[17] J. Li, R.V. Tappero, A.S. Acerbo, H. Yan, Y. Chu, G.V. Lowry, J.M. Unrine, *Env. Sci Nano* **2019**, *6*, 273.

[18] Y. Dan, X. Ma, W. Zhang, K. Liu, C. Stephan, H. Shi, *Anal. Bioanal. Chem.* **2016**, *408*, 5157.

[19] P. Chaurand, W. Liu, D. Borschneck, C. Levard, M. Auffan, E. Paul, B. Collin, I. Kieffer, S. Lanone, J. Rose, J. Perrin, *Sci. Rep.* **2018**, *8*, 4408.

[20] J. Modrzynska, T. Berthing, G. Ravn-Haren, K. Kling, A. Mortensen, R.R. Rasmussen, E.H. Larsen, A.T. Saber, U. Vogel, K. Loeschner, *PloS One* **2018**, *13*, e0202477.

[21] J.A. Hernandez-Viezcas, H. Castillo-Michel, J.C. Andrews, M. Cotte, C. Rico, J.R. Peralta-Videa, Y. Ge, J.H. Priester, P.A. Holden, J.L. Gardea-Torresdey, *ACS Nano* **2013**, *7*, 1415.

[22] L. Veith, D. Dietrich, A. Vennemann, D. Breitenstein, C. Engelhard, U. Karst, M. Sperling, M. Wiemann, B. Hagenhoff, *J. Anal. Spectrom.* **2018**, *33*, 491.

[23] M.P.S. Idelchik, J. Dillon, L. Abariute, M.A. Guttenberg, A. Segarceanu, N.M. Neu-Baker, S.A. Brenner, *J. Microsc.* **2018**, *271*, 69.

[24] Q. Li, Z. Wang, J. Mo, G. Zhang, Y. Chen, C. Huang, *Sci. Rep.* **2017**, *7*, 2965.

[25] J.S. Souris, C.-H. Lee, S.-H. Cheng, C.-T. Chen, C.-S. Yang, J.A. Ho, C.-Y. Mou, L.-W. Lo, *Biomaterials* **2010**, *31*, 5564.

[26] D. Drescher, I. Zeise, H. Traub, P. Guttmann, S. Seifert, T. Büchner, N. Jakubowski, G. Schneider, J. Kneipp, *Adv. Funct. Mater.* **2014**, *24*, 3765.

[27] R. Popovtzer, A. Agrawal, N.A. Kotov, A. Popovtzer, J. Balter, Thomas.E. Carey, R. Kopelman, *Nano Lett.* **2008**, *8*, 4593.

[28] A.S. Thakor, R. Luong, R. Paulmurugan, F.I. Lin, P. Kempen, C. Zavaleta, P. Chu, T.F. Massoud, R. Sinclair, S.S. Gambhir, *Sci. Transl. Med.* **2011**, *3*, 79ra33.

[29] T. Liu, I. Kempson, M. de Jonge, D.L. Howard, B. Thierry, *Nanoscale* **2014**, *6*, 9774.

[30] I.M. Rio-Echevarria, J. Ponti, A. Bogni, D. Gilliland, M. Altissimo, L. Pascolo, G. Ceccone, A. Gianoncelli, *X-Ray Spectrom.* **2019**, *48*, 94.

[31] L. Veith, A. Vennemann, D. Breitenstein, C. Engelhard, M. Wiemann, B. Hagenhoff, *Analyst* **2017**, *142*, 2631.

[32] A. Goldstein, Y. Soroka, M. Frusic-Zlotkin, I. Popov, R. Kohen, *J. Microsc.* **2014**, *256*, 237.

[33] K. Yamashita, Y. Yoshioka, K. Higashisaka, K. Mimura, Y. Morishita, M. Nozaki, T. Yoshida, T. Ogura, H. Nabeshi, K. Nagano, Y. Abe, H. Kamada, Y. Monobe, T. Imazawa, H. Aoshima, K. Shishido, Y. Kawai, T. Mayumi, S. Tsunoda, N. Itoh, T. Yoshikawa, I. Yanagihara, S. Saito, Y. Tsutsumi, *Nat. Nanotechnol.* **2011**, *6*, 321.

[34] D.J. Graham, J.T. Wilson, J.J. Lai, P.S. Stayton, D.G. Castner, *Biointerphases* **2015**, *11*, 02A304.

[35] B. Kann, H.L. Offerhaus, M. Windbergs, C. Otto, *Adv. Drug Deliv. Rev.* **2015**, *89*, 71.

[36] Z. Liu, C. Davis, W. Cai, L. He, X. Chen, H. Dai, *Proc. Natl. Acad. Sci.* **2008**, *105*, 1410.

[37] L. Carlred, A. Gunnarsson, S. Solé-Domènech, B. Johansson, V. Vukojević, L. Terenius, A. Codita, B. Winblad, M. Schalling, F. Höök, P. Sjövall, *J. Am. Chem. Soc.* **2014**, *136*, 9973.

[38] S. Vanden-Hehir, W.J. Tipping, M. Lee, V.G. Brunton, A. Williams, A.N. Hulme, *Nanomater. Basel Switz.* **2019**, *9*, 341.
